# Integration of EpiSign, facial phenotyping, and likelihood ratio interpretation of clinical abnormalities in the re‐classification of an *ARID1B* missense variant

**DOI:** 10.1002/ajmg.c.32056

**Published:** 2023-08-31

**Authors:** Caitlin Forwood, Katie Ashton, Ying Zhu, Futao Zhang, Kerith‐Rae Dias, Krystle Standen, Carey‐Anne Evans, Louise Carey, Michael Cardamone, Carolyn Shalhoub, Hala Katf, Carlos Riveros, Tzung‐Chien Hsieh, Peter Krawitz, Peter N Robinson, Tracy Dudding‐Byth, Bekim Sadikovic, Jason Pinner, Michael F. Buckley, Tony Roscioli

**Affiliations:** ^1^ NSW Health Pathology Randwick Genomics Prince of Wales Hospital Sydney Australia; ^2^ Centre for Clinical Genetics Sydney Children's Hospital Randwick Australia; ^3^ Neuroscience Research Australia (NeuRA) University of New South Wales Sydney Australia; ^4^ Sydney Children's Hospital Randwick Australia; ^5^ School of Women's and Children's Health UNSW Sydney Australia; ^6^ Bioinformatics, Hunter Medical Research Institute Newcastle Australia; ^7^ Institute for Genomic Statistics and Bioinformatics University Hospital Bonn Bonn Germany; ^8^ JAX Center for Precision Genetics The JAX Cancer Center Farmington Connecticut USA; ^9^ Genetics of Learning Disability (GoLD) Service Waratah Australia; ^10^ London Health Sciences Centre, Verspeeten Clinical Genome Centre Western University London Canada; ^11^ School of Clinical Medicine UNSW Sydney Australia

**Keywords:** ARID1B, Coffin–Siris syndrome, episignature, FaceMatch, GestaltMatcher, LIRICAL

## Abstract

Heterozygous *ARID1B* variants result in Coffin–Siris syndrome. Features may include hypoplastic nails, slow growth, characteristic facial features, hypotonia, hypertrichosis, and sparse scalp hair. Most reported cases are due to *ARID1B* loss of function variants. We report a boy with developmental delay, feeding difficulties, aspiration, recurrent respiratory infections, slow growth, and hypotonia without a clinical diagnosis, where a previously unreported *ARID1B* missense variant was classified as a variant of uncertain significance. The pathogenicity of this variant was refined through combined methodologies including genome‐wide methylation signature analysis (EpiSign), Machine Learning (ML) facial phenotyping, and LIRICAL. Trio exome sequencing and EpiSign were performed. ML facial phenotyping compared facial images using FaceMatch and GestaltMatcher to syndrome‐specific libraries to prioritize the trio exome bioinformatic pipeline gene list output. Phenotype‐driven variant prioritization was performed with LIRICAL. A de novo heterozygous missense variant, *ARID1B* p.(Tyr1268His), was reported as a variant of uncertain significance. The ACMG classification was refined to likely pathogenic by a supportive methylation signature, ML facial phenotyping, and prioritization through LIRICAL. The *ARID1B* genotype–phenotype has been expanded through an extended analysis of missense variation through genome‐wide methylation signatures, ML facial phenotyping, and likelihood‐ratio gene prioritization.

## 1 INTRODUCTION

*ARID1B* is a frequent cause of intellectual disability (ID; van der Sluijs et al., 2019). *ARID1B*‐related neurodevelopmental disorder (*ARID1B*‐RD) displays phenotypic heterogeneity, including Coffin–Siris syndrome 1, OMIM #135900 (ARID1B‐CSS) and ID with or without dysmorphic features (Vergano et al., 1993). *ARID1B*‐RD can present with variable ID, myopia, spasticity, epilepsy, feeding difficulties, and laryngomalacia, while typical features of ARID1B‐CSS also include nail hypoplasia, hypotonia, limb hypertrichosis, and characteristic facial features (van der Sluijs et al., 2019; Vergano et al., 1993). The clinical features observed in *ARID1B* cohorts depend on the selection criteria. Due to ascertainment bias, individuals with a priori diagnosis of ARID1B‐CSS may over‐represent more discernible features (Bogershausen & Wollnik, 2018; Santen et al., 2014; van der Sluijs et al., 2019; Vergano et al., 1993). Pathogenic variants may be inherited from a mildly affected parent (Mignot et al., 2016; van der Sluijs et al., 2019, 2021). BAF complex disruption is associated with Coffin–Siris and Nicolaides–Baraitser syndromes (*SMARCA2* OMIM #600014; Wolff et al., 2012), collectively known as BAFopathies, which include developmental delay, coarse facial features, and phalangeal abnormalities (Aref‐Eshghi et al., 2018, 2019). Coffin–Siris syndrome may result from pathogenic variants in other genes, including *ARID1A* (CSS2 OMIM #614607), *SMARCB1* (CSS3 OMIM #614608), *SMARCA4* (CSS4 OMIM #614609), *SMARCE1* (CSS5 OMIM #616938), *ARID2* (CSS6 OMIM #617808), *DPF2* (CSS7 OMIM #618027), *SMARCC2* (CSS8 OMIM #618362), and *SMARCD1* (CSS11 OMIM #618779).

*ARID1B* loss of function variants are identified commonly in *ARID1B*‐RD. Missense variants are reported rarely and are usually considered not to be pathogenic (Santen et al., 2014; van der Sluijs et al., 2019). Previous reports of missense *ARID1B* variants include a maternally inherited heterozygous variant c.6092 T > C p.(Ile2031Thr) in a proband with agenesis of the corpus callosum where the mother had mild ID (de novo in mother; Mignot et al., 2016). A likely pathogenic missense variant has been reported in a male with ID and facial dysmorphism, c.2308G > A p.(Gly770Arg; Grozeva et al., 2015). Other published *ARID1B* missense variants include two inherited and two de novo variants presenting with short stature (Yu et al., 2015) and two de novo variants in children with ID (Zhang et al., 2020). At the time of reporting, ClinVar documents 18 *ARID1B* likely pathogenic and 2 pathogenic missense variants in *ARID1B* (see Table S1) compared to 123 truncating variants. Additional *ARID1B* missense variants in ClinVar include 400 variants of uncertain significance, 125 likely benign, 49 benign, and 47 variants with conflicting interpretations (https://www.ncbi.nlm.nih.gov/clinvar/?term=ARID1B%5Bgene%5D&redir=gene).

We describe a 1‐year‐old male with moderate ID, hypotonia, feeding difficulties, nephrolithiasis, aspiration, and recurrent lower respiratory tract infections. A heterozygous *ARID1B* missense variant identified via trio whole exome sequencing was reported as a variant of uncertain significance (VUS). The combination of genome‐wide methylation signatures, ML Facial Phenotyping (GestaltMatcher and FaceMatch), and likelihood ratio interpretation of clinical abnormalities (LIRICAL) variant reprioritization showed utility in pathogenicity reclassification to obtain a refined diagnosis of likely pathogenic.

### 1.1 Case report

The proband was born at 30 weeks' gestation with growth parameters adjusted for prematurity showing weight 1.63 kg (80th centile), length 40.5 cm (42nd centile), and head circumference 30.0 cm (95th centile). His parents were non‐consanguineous. He was referred at 9 weeks corrected gestational age (CGA) to assess central apneas, laryngomalacia, an atrial septal defect, and feeding difficulties with slow growth. His weight was 4.2 kg (15th centile), length 53 cm (7th centile), and head circumference of 40 cm (69th centile). He was observed to have coarse facial features, bitemporal narrowing, dolichocephaly, and apparent rhizomelia. An uncoordinated suck and hyperekplexia with normal reflexes and tone were observed. A diagnosis of nephrolithiasis was made based on echogenic material on renal ultrasound with subsequent hypercalciuria on urinary studies. Based on a maternal history of renal calculi, fluids optimization was recommended, and potassium citrate commenced. A skeletal survey, MRI brain, SNP‐microarray, and biochemical testing, including investigations for congenital disorders of glycosylation and storage disorders, were non‐diagnostic. On review at 8 months CGA, he had moderate to severe developmental delay, ongoing feeding difficulties, right‐sided conductive hearing loss, severe gastro‐esophageal reflux, and dysphagia requiring gastrostomy feeds. Supplemental oxygen was needed for central apneas. His weight was 9.5 kg (65th centile), length 65 cm (32nd centile), and head circumference 48.2 cm (>99th centile). He had nonfamilial facial features with full lips, ptosis, a short full nose, and a prominent glabella. Deep palmar and sole creases, hypodontia, and hypotonia were present. Repeat MRI brain showed mild prominence of the ventricles and extra‐axial CSF space, considered within normal limits for age and a likely small arachnoid cyst in the left posterior fossa. There was no other significant intracranial structural abnormality, and a specific clinical diagnosis was not made.

## 2 MATERIALS AND METHODS

DNA was extracted from peripheral blood, and exome sequencing was performed using CRE II whole exome kit (Agilent) on a NovaSeq 6000 instrument (Illumina) with variant annotation, filtering, and prioritization were performed using an in‐house genomics pipeline, Genomics Annotation and Interpretation Application (GAIA) as described previously (Sundercombe et al., 2021). Variants were prioritized using an automated PubMed search linked to the referral phenotype performed within GAIA as well as annotation from clinical genetic and variant databases, including ClinVar (http://www.ncbi.nlm.nih.gov/clinvar), HGMD (http://www.hgmd.cf.ac.uk/ac/index.php), and OMIM (http://www.omim.org).

Genome‐wide methylation testing was performed on bisulfite converted DNA using the EZ DNA Methylation Kit (Zymo Research). Following conversion, methylation analysis was performed using the Infinium MethylationEPIC beadchip array (Illumina) and processed on a NextSeq 550 (Illumina). The resulting methylated and unmethylated signal intensity data were imported into R 3.5.2 for analysis. Normalization was performed according to the Illumina normalization method with background correction using the minfi package. The methylation level for each probe was measured as a beta value calculated from the ratio of the methylated signals to the sum of unmethylated and methylated signals (Aref‐Eshghi et al., 2019). DNA methylation signature analysis was performed using the EpiSign analysis protocol as previously described (Sadikovic et al., 2021).

FaceMatch (www.facematch.org.au; Dudding‐Byth et al., 2017) and GestaltMatcher (www.gestaltmatcher.org; Hsieh et al., 2022; Hustinx et al., 2023) were utilized for ML facial phenotyping to compare facial images and gene lists generated from the genomic output from GAIA after trio exome analysis. Weighted facial matches were assessed compared to the clinical diagnosis in the proband.

LIRICAL (https://lirical.readthedocs.io/en/latest/; Robinson et al., 2020) was utilized to perform phenotype‐driven prioritization of candidate genes. The phenotypic features were standardized to the Human Phenotype Ontology (HPO) terms and used to reprioritize the list of filtered variants from the genomic pipeline.

## 3 RESULTS

### 3.1 Variant curation

Variant curation was phenotype‐driven with classification according to the American College of Medical Genetics and Genomics (ACMG) criteria. Trio exome sequencing (ES) identified a de novo heterozygous variant predicted to result in a missense change in *ARID1B*: Chr6(GRCh38) g.157184318 T > C, NM_001374828.1:c.3802 T > C, p.(Tyr1268His) in exon 14 of 21 where the normally encoded tyrosine is replaced by histidine. The pathogenicity categorization for the variant was initially assessed as a VUS.

The *ARID1B*:p.(Tyr1268His) variant has not been reported in the literature and is absent in population databases (gnomAD) [PM2]. While *ARID1B* is not uniformly under missense constraint (Z = 2.59, o/e = 0.79[0.75–0.83]), there is a localized missense constraint of the region containing the variant in the ARID/BRIGHT DNA binding domain (PF01388) with a MetaDome tolerance score of dn/ds = 0.1 (highly intolerant to missense change; see Figure S1a; Wiel et al., 2019). There is a cluster of likely pathogenic or pathogenic variants in exon 14 with an absence of benign missense variation [PM1] (Figure S1b). Protein prediction modeling using the Protein Hope platform showed that the mutant histidine variant was smaller and less hydrophobic than the native tyrosine. In silico prediction tools supported a deleterious effect [PP3_moderate] (Mutscore 0.968, VARITY_R 0.89, ClinPred 0.9993, REVEL 0.891, Varcards 21:23). Missense variants are uncommonly reported as pathogenic or likely pathogenic in *ARID1B‐RD* [BP1].

Given the phenotypic correlation with ARID1B‐CSS, the PS2_supporting criterion was included in the pathogenicity categorization, downgraded due to the significant genetic heterogeneity of ID. Using the ACMG classification criteria for pathogenicity categorization, the de novo missense *ARID1B*:p.(Tyr1268His) variant reported as a VUS with a posterior probability of pathogenicity of 67.5% based on one moderate [PM1], three supporting [PM2_supporting, PS2_supporting, and PP3] and one benign supporting criteria [BP1] (Tavtigian et al., 2018).

Additional independent phenotyping was performed using EpiSign, ML facial technology, and LIRICAL to determine whether the Bayesian components of phenotype specificity or the number of genes to be considered could be altered in the ACMG criteria.

### 3.2 Episignature

**FIGURE 1:**
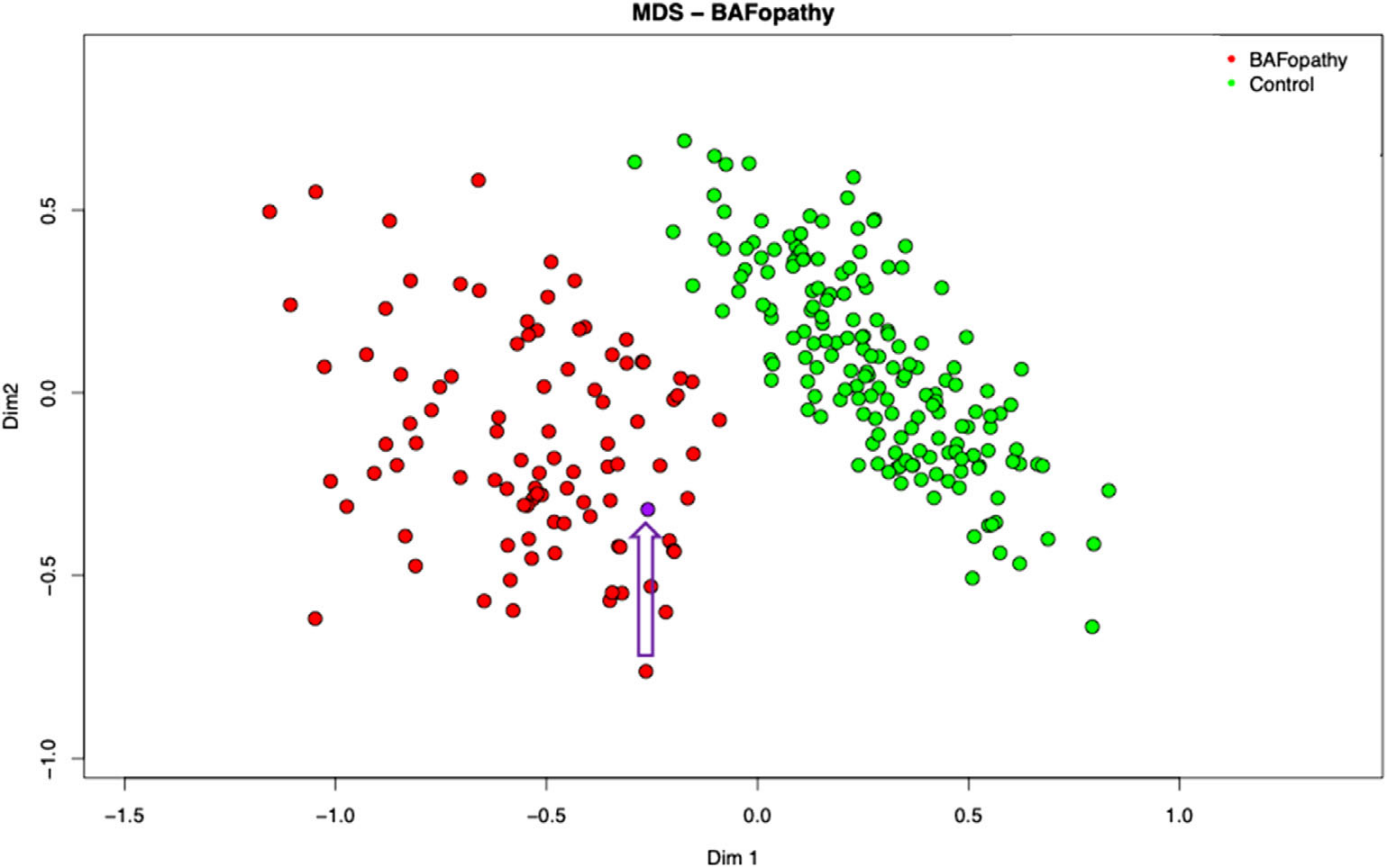
Episignature image, proband in purple with arrow, BAFopathy positive episignatures in red.

Mutations in *ARID1B*, and several other genes that are part of the BAF complex, result in a distinct DNA methylation profile in patients with BAF‐complex disorders (Aref‐Eshghi et al., 2018). This episignature shows a strong association with high sensitivity and specificity for BAF‐complex disorders, allowing its use as a diagnostic biomarker. The biological causation has not been precisely determined. However, one plausible explanation is disruption in a transcriptional regulatory complex leads to disruptions in gene expression and chromatin states during early development that remain detectable in differentiated tissues in affected patients. The methylation profile was consistent with a BAFopathy episignature (Aref‐Eshghi et al., 2018), with the variant clustering with other individuals with BAFopathies (see Figure 1). The BAF episignature cohort is genetically heterogeneous including both truncating and pathogenic missense variants(Aref‐Eshghi et al., 2018). There is insufficient evidence currently of sub clustering based on the variant type within each gene. This result reduced the genetic heterogeneity for the de novo classification PS2, allowing upgrading the strength of PS2_supporting to PS2_moderate, as there are currently approximately 90 genes with an episignature, and no additional variants in BAFopathy genes were identified.

### 3.3 ML‐facial phenotyping

**FIGURE 2:**
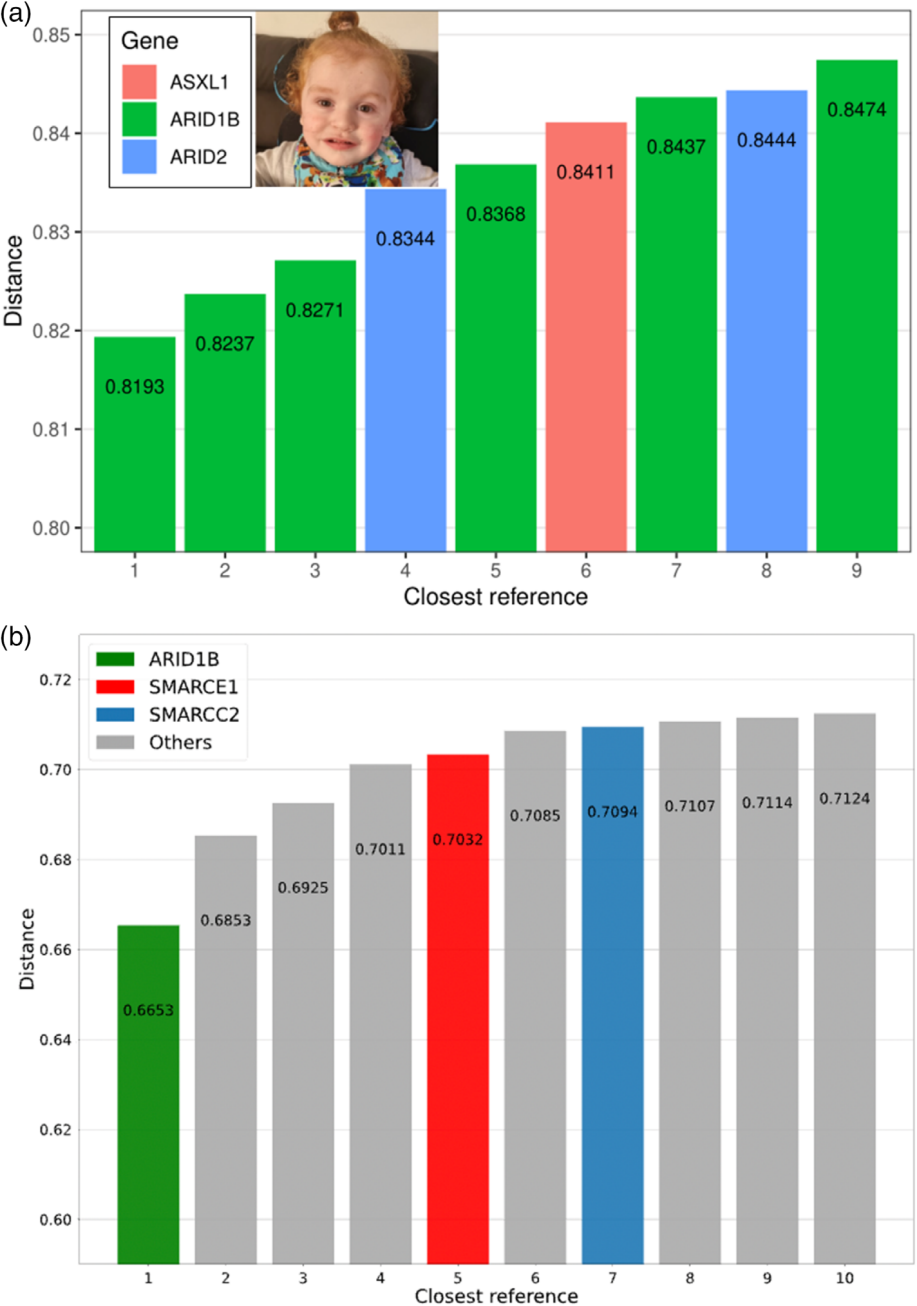
(a) Results from FaceMatch, rank results indicate the order in increasing distance, and each bar match to a reference image within the 67 reference individuals in the FaceMatch database with OMIM syndrome and gene in the list of interest. The number of reference matches with distance below the threshold of 0.85. (b) Results from GestaltMatcher: rank results to the top‐10 similar patients among 7459 patients' images with 449 different disorders in GestaltMatcher Database. The ranks of patients' genes are *ARID1B*, *ZBTB20*, *MTOR*, *KCNK9*, *SMARCE1*, *RAC1*, *SMARCC2*, *MAP3K7*, *ACTG1*, and *CREBBP*.

FaceMatch produces a similarity score to a series of photographs converted to a distance via a linear transformation and ranked. Distances above 0.85 are non‐informative, 0.8 to 0.85 indicate a mild similarity, and below 0.8 is a possible match. The face alone did not generate a FaceMatch correlation; however, when filtered against only the filtered genes from the trio ES, *ARID1B* was calculated to be the top‐ranked gene with a distance of 0.8193 (see Figure 2a).

GestaltMatcher uses deep‐learning algorithms to quantify facial image similarities to 7459 patients' images with 449 different disorders in GestaltMatcher Database (GMDB; Lesmann et al., 2023). In GMDB, there were 130 images with CSS. *ARID1B* was GestaltMatcher's top prioritized gene for the described individual (see Figure 2b). Therefore, *ARID1B* was the only matched possibility in the genomic pipeline‐filtered genes. Based on these results, the pathogenic supporting term for a consistent phenotype PP4 was added.

### 3.4 LIRICAL

LIRICAL uses a likelihood ratio (LR) to estimate the posttest diagnostic probability, the LR for each observed HPO phenotype, and the predicted pathogenicity of observed genotypes (Robinson et al., 2020). The referral terms chosen were hypotonia (HP:0001252), atrial septal defect (HP:0001631), postnatal growth retardation (HP:0008897), aspiration (HP:0002835), recurrent infections due to aspiration (HP:0004891), feeding difficulties in infancy (HP:0008872), and moderate global developmental delay (HP:0011343). LIRICAL identified *ARID1B* as the top‐ranked gene in the exome sequencing output with a posttest probability of 96%. This prioritization increased the Bayesian contribution of phenotype and genotype, further supporting pathogenicity.

## 4 RECLASSIFICATION

The revised phenotypic correlation included the pathogenic supporting term for a consistent phenotype [PP4] and upgrading of PS2 [PS2_moderate]. After the initial reporting of the case, based on weighting recommendations for PP3, due to a REVEL score of 0.891, PP3 was upgraded to [PP3_moderate] (Pejaver et al., 2022). The de novo missense *ARID1B* variant was reclassified as likely pathogenic with a posterior probability of pathogenicity of 97.5% based on three moderate [PM1, PP3_moderate, and PS2_moderate] and three supporting criteria [PM2_supporting, PP2, and PP4]. BP1 was used given the predominant LOF *ARID1B* mutational mechanism (Pejaver et al., 2022).

## 5 DISCUSSION

This report highlights the challenges in assigning pathogenicity to de novo missense variants in a gene typically characterized by LOF. *ARID1B* haploinsufficiency could result from missense variants, although variants within exon 1 are unlikely to impact protein function due to frequent benign missense variation in that region (Bogershausen & Wollnik, 2018). Published likely pathogenic missense variants typically cluster near the C‐terminus (Bogershausen & Wollnik, 2018). Many *ARID1B* missense variants cluster near the highly conserved missense variant identified in this study (Grozeva et al., 2015; Mignot et al., 2016; Quinodoz et al., 2022). Classifying a novel *ARID1B* missense variant as likely pathogenic demonstrates the importance of considering missense variation in diseases typically associated with LOF or haploinsufficiency.

Clinical phenotyping shows ascertainment bias, with extreme phenotypes facilitating both syndrome recognition and gene identification. Many syndromes have facial dysmorphism as key diagnostic features; however, reliance on clinical diagnosis biases means that traditional phenotyping provides a weak ACMG framework Bayesian likelihood modification. ML‐facial phenotyping can increase the specificity of facial phenotyping (Dudding‐Byth et al., 2017). GestaltMatcher and FaceMatch can match individuals with known or unknown disorders with functionality to explore facial similarity (Dudding‐Byth et al., 2017; Hsieh et al., 2022). Syndrome distinctiveness in GestaltMatcher correlated with expert opinion (Hsieh et al., 2022). Similarly, the diagnostic rate was >80% for all syndromes in the FaceMatch pilot study and diagnostically more accurate than three senior clinical geneticists (*p* < 0.00001; Dudding‐Byth et al., 2017).

Human‐independent phenotyping methodologies provide non‐biased syndrome correlation to facilitate the interpretation of DNA sequencing data (Dudding‐Byth et al., 2017; Hsieh et al., 2022), with a role in classifying *ARID1B* variants (van der Sluijs et al., 2021). Both GestaltMatcher and FaceMatch had *ARID1B* as the top‐ranked gene in the variants from the genomic output. These results are consistent with facial phenotyping providing the highest diagnostic accuracy when combined with a filtered gene list.

Genome‐wide DNA methylation patterns may provide additional evidence for variant classification in genes affected by chromatin remodeling or epigenetic reprogramming (Aref‐Eshghi et al., 2019; Sadikovic et al., 2021; Rooney & Sadikovic, 2022). EpiSign analysis was particularly important for reclassification as it provided an independent functional link between an atypical *ARID1B* missense variant and a cluster of *ARID1B* episignatures.

The correct diagnosis was within the LIRICAL first three ranks in 92.9% of 262 Mendelian diseases, with a mean posttest probability of 67.3% (Robinson et al., 2020). The HPO terms used are identified commonly in many syndromes, but when applied to a filtered gene list, they became highly specific. Global developmental delay, significant gastro‐esophageal reflux, and feeding difficulties are frequent in ID and CSS (van der Sluijs et al., 2019; Vergano et al., 1993); however, none of the other gene variants considered from the exome analysis scored on these phenotypes. While the nephrolithiasis and hypercalciuria were considered familial in nature, nephrolithiasis are also reported in *ARID1B* patients (van der Sluijs et al., 2019). Although hypertrichosis, sparse scalp hair, and fifth‐digit hypoplasia were not observed in the proband, full lips were consistent with *ARID1B*‐CSS, adding Bayesian support for the pathogenicity of the variant.

## 6 CONCLUSION

The *ARID1B* variant has been re‐classified through an extended analysis, providing a diagnosis and accurate reproductive counseling. EpiSign, ML‐facial phenotyping, and LIRICAL demonstrated the utility of methylation signatures, phenotyping independent of clinical assessments, and the reprioritization of variants from a genomic pipeline for variant reclassification. Identifying an *ARID1B* VUS should prompt discussion with the referring clinician about the possibility of an *ARID1B‐RD* and consider further investigation using phenotyping and episignature analyses.

## AUTHOR CONTRIBUTIONS

**Caitlin Forwood**: conceptualization contributed clinical background, original draft, and revisions. **Jason Pinner**: lead clinician contributing to case report, conceptualization, manuscript revision. **Katie Ashton**: genomic analysis and reporting, manuscript revision. **Ying Zhu** and **Futao Zhang**: bioinformatic pipeline development, data analysis and integration with LIRICAL. **Kerith‐Rae Dias, Krystle Standen, Carey‐Anne Evans**, and **Louise Carey**: data analysis and application of EpiSign. **Michael Cardamone**, **Carolyn Shalhoub**, and **Hala Katf**: clinicians who contributed clinical information. **Carlos Rivera** and **Tracy Dudding‐Byth**: development of FaceMatch and yielded results with the genomic output, manuscript revision. **Tzung‐Chien Hsieh** and **Peter Krawitz**: development of GestaltMatcher and yielded results with the genomic output, manuscript revision. **Peter Robinson**: designed LIRICAL and yielded the results with the genomic output. **Bekim Sadikovic**: development of EpiSign, results interpretation/application and manuscript revision. **Michael Buckley** and **Tony Roscioli**: conceptualization, supervision of data and manuscript, genomic analysis, and data analysis/integration. All co‐authors reviewed and revised the manuscript.

## CONFLICT OF INTEREST STATEMENT

The authors declare no conflicts of interest.

## Supporting information


**FIGURE S1: (**a) Tyrosine 1185 residue MetaDome tolerance score of dn/ds = 0.1 (highly intolerant to missense change) (b) Screenshot from Mutscore with pathogenic/likely pathogenic variants from ClinVar shown in yellow and a whole of exon 13 with an absence of benign missense variation (note different transcript used NM_001346813.1) (c) Hidden Markov Model logogram displaying conservation as a graphical representation based on the percentage height that an amino acid occupies. Red vertical lines are places where the sequences poorly align or gaps in the sequence. The large Y represents Tyrosine and supports the evolutionary conservation of the amino acid.

**TABLE S1:** ClinVar LP/P missense variants.

## Data Availability

The data that support the findings of this study are available on request from the corresponding author. The data are not publicly available due to privacy or ethical restrictions.
