# Supplementary material for: Integration of EpiSign, facial phenotyping, and likelihood ratio interpretation of clinical abnormalities in the re‐classification of an ARID1B missense variant
Source: Am J Med Genet C Semin Med Genet. 2023 Aug 31;193(3):e32056. doi: 10.1002/ajmg.c.32056 (PMC10952833; doi:10.1002/ajmg.c.32056)
Supplement: Supplementary file 1 — FIGURE S1: (a) Tyrosine 1185 residue MetaDome tolerance score of dn/ds = 0.1 (highly intolerant to missense change) (b) Screenshot from Mutscore with pathogenic/likely pathogenic variants from ClinVar shown in yellow and a whole of exon 13 with an absence of benign missense variation (note different transcript used NM_001346813.1) (c) Hidden Markov Model logogram displaying conservation as a graphical representation based on the percentage height that an amino acid occupies. Red vertical lines are places where the sequences poorly align or gaps in the sequence. The large Y represents Tyrosine and supports the evolutionary conservation of the amino acid. [file AJMG-193-0-s001.docx]

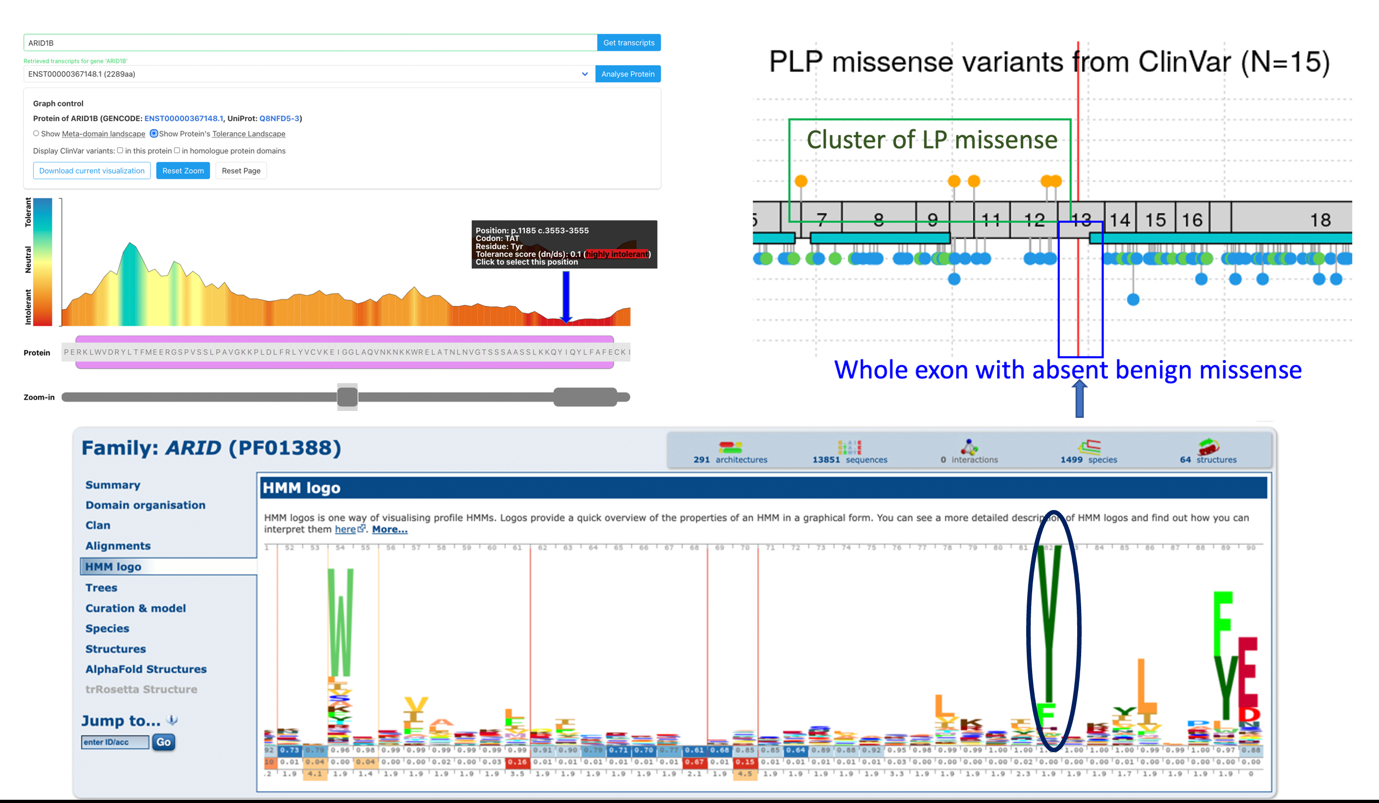


**Supplementary Figure 1**

1a) Tyrosine 1185 residue MetaDome tolerance score of dn/ds=0.1 (highly intolerant to missense change) 1b) Screenshot from Mutscore with pathogenic/likely pathogenic variants from ClinVar shown in yellow and a whole of exon 13 with an absence of benign missense variation (note different transcript used NM_001346813.1) 1c) Hidden Markov Model logogram displaying conservation as a graphical representation based on the percentage height that an amino acid occupies. Red vertical lines are places where the sequences poorly align or gaps in the sequence. The large Y represents Tyrosine and supports the evolutionary conservation of the amino acid.
