# Supplementary material for: Integration of EpiSign, facial phenotyping, and likelihood ratio interpretation of clinical abnormalities in the re‐classification of an ARID1B missense variant
Source: Am J Med Genet C Semin Med Genet. 2023 Aug 31;193(3):e32056. doi: 10.1002/ajmg.c.32056 (PMC10952833; doi:10.1002/ajmg.c.32056)
Supplement: Supplementary file 2 — TABLE S1: ClinVar LP/P missense variants. [file AJMG-193-0-s002.docx]

**Supplementary Table 1: ClinVar LP/P missense variants**

| **Name** | **Gene(s)** | **Condition(s)** | **Clinical significance** | **Review status** | **Accession** |
| --- | --- | --- | --- | --- | --- |
| c.266G>A p.(Gly89Asp) | *ARID1B* | ARID1B-related BAFopathy\|Coffin-Siris syndrome 1 | LP | single submitter | VCV000374256 |
| c.1651C>A p.(Gln551Lys) | *ARID1B* | ARID1B-related BAFopathy | LP | single submitter | VCV001177346 |
| c.2581G>A p.(Gly861Ser) | *ARID1B* | ARID1B-related BAFopathy\|not provided\|Coffin-Siris syndrome 1 | LP | multiple submitters, no conflicts | VCV000372882 |
| c.2762G>T p.(Gly921Val) | *ARID1B* | Coffin-Siris syndrome 1 | P | single submitter | VCV001527910 |
| c.3258G>T p.(Met1086Ile) | *ARID1B* | not provided | LP | single submitter | VCV000372900 |
| c.3345G>C p.(Lys1115Asn) | *ARID1B* | Coffin-Siris syndrome 1 | P | no assertion criteria provided | VCV001174076 |
| c.3665A>T p.(Asp1222Val) | *ARID1B* | not provided | LP | single submitter | VCV000545058 |
| c.3704G>A p.(Gly1235Asp) | *ARID1B* | not provided | LP | single submitter | VCV000218776 |
| c.5896C>T p.(Arg1966Cys) | *ARID1B* | Coffin-Siris syndrome 1 | LP | no assertion criteria provided | VCV001184820 |
| c.6263T>C p.(Met2088Thr) | *ARID1B* | Coffin-Siris syndrome 1 | LP | multiple submitters, no conflicts | VCV000689678 |
| c.6461T>C p.(Ile2154Thr) | *ARID1B* | not provided | LP | single submitter | VCV001254408 |
| c.6464C>T p.(Ser2155Phe) | *ARID1B* | not provided | LP | single submitter | VCV000265517 |
| c.6493G>A p.(Glu2165Lys) | *ARID1B* | not provided | LP | single submitter | VCV000420932 |
| c.6617T>C p.(Leu2206Pro) | *ARID1B* | Coffin-Siris syndrome 1 | LP | single submitter | VCV000974620 |
| c.6775T>C p.(Ser2259Pro) | *ARID1B* | not provided | LP | single submitter | VCV000384093 |
